# Immunisation with the membrane proximal external region of gp41 of HIV-1 grafted into the transmembrane envelope protein of a gammaretrovirus

**DOI:** 10.1186/1742-4690-9-S2-P308

**Published:** 2012-09-13

**Authors:** N Strasz, V Morozov, J Kreutzberger, M Lau, J Denner

**Affiliations:** 1 Robert Koch Institute, Berlin, Germany

## Background

Immunisation with the transmembrane envelope (TM) proteins p15E of different gammaretroviruses (e.g., porcine endogenous retrovirus, feline leukaemia virus, Koala retrovirus) resulted in strong neutralising activity, the antibodies recognised epitopes in the fusion peptide proximal region (FPPR) and in the membrane proximal external region (MPER). The MPER epitopes were localised similarly as the epitopes recognised by the broadly neutralising antibodies 2F5 and 4E10 in gp41 of HIV-1. Despite the evolutionary difference between HIV-1 and the gammaretroviruses, the MPER epitope of antibodies neutralising PERV (FEGWFN) showed partial homology to the epitope of the 4E10 (NWFNIT, note three identical amino acids). To generate hybrid antigens able to induce 2F5/4E10-like antibodies, sequences of the MPER and FPPR of gp41 were grafted into the p15E backbone of a gammaretrovirus.

## Methods

Different hybrid antigens were cloned, expressed in E. coli and purified. Immunisation studies in rats and guinea pigs were performed and the antisera were characterised by ELISA, Western blot analysis, epitope mapping using microarray chips with overlapping peptides and a neutralisation assay based on TZM-bl cells.

## Results

Antibodies against gp41 of HIV-1 were induced, recognising epitopes in the FPPR, but also the 2F5 epitope (ELDKWA) in the MPER. Step by step changes in the sequence of the hybrids resulted in improved binding of the antibodies to this epitope. However, none of the immune sera or purified IgG neutralised HIV-1 more that 50%.

## Conclusion

Since modifications in the hybrid proteins led to an increased anti-MPER response, it may be expected that further modifications increase neutralisation efficacy and that these hybrids may be the basis for candidate vaccines against HIV-1.

Performed in the frame of the EuroNeut-41 project in the Seventh Framework Program.

